# Comparison of Circadian Efficacy of Tafluprost Eye Drops (Taflotan Sine^®^) with Latanoprost Eye Drops (Xalatan^®^) in the Treatment of Open-Angle Glaucoma and Ocular Hypertension

**DOI:** 10.3390/jcm14248932

**Published:** 2025-12-17

**Authors:** Anna Beck, Katharina Theresa Rauschkolb-Olk, Anna Maria Welzel, Peter Wolfrum, Christina Korb, Irene Schmidtmann, Elsa Wilma Boehm, Katrin Lorenz

**Affiliations:** 1 Department of Ophthalmology, University Medical Center, Johannes Gutenberg-University, 55131 Mainz, Germany; katharina.rauschkolb@unimedizin-mainz.de (K.T.R.-O.); annamaria.welzel@unimedizin-mainz.de (A.M.W.); peter.wolfrum@unimedizin-mainz.de (P.W.); christina.korb@unimedizin-mainz.de (C.K.); elsawilma.boehm@unimedizin-mainz.de (E.W.B.); katrin.lorenz@unimedizin-mainz.de (K.L.); 2 Institute for Medical Biostatistics, Epidemiology and Informatics, University Medical Center, Johannes Gutenberg-University, 55131 Mainz, Germany; irene.schmidtmann@uni-mainz.de

**Keywords:** glaucoma, preservative-free tafluprost, latanoprost, diurnal intraocular pressure measurement

## Abstract

**Background/Objectives:** To investigate the non-inferiority of preservative-free (PF) tafluprost eye drops (Taflotan sine^®^) compared to latanoprost eye drops (Xalatan^®^) in reducing mean 24 h intraocular pressure (IOP) on the second day of a diurnal IOP measurement. **Methods:** In this retrospective monocentric cohort study, patients suffering from primary open-angle glaucoma, ocular hypertension, normal tension glaucoma, pigment dispersion glaucoma, or pseudoexfoliation glaucoma who had undergone inpatient diurnal IOP measurement were included. Patients of cohort 1 used latanoprost eye drops as monotherapy; patients of cohort 2 used preservative-free tafluprost eye drops. Data from 7 January 2005 to 9 July 2019, inclusive, were analyzed. **Results:** Ninety-three eyes were included (n = 59 latanoprost group, n = 34 PF tafluprost group). Mean 24 h IOP on day 2 was 14.1 mmHg (SD 2.3) in the latanoprost group and 14.5 mmHg (SD 3.4) in the PF tafluprost group. The non-inferiority of PF tafluprost eye drops to latanoprost eye drops in efficacy on mean 24 h IOP could not be confirmed (95% CI −1.5, ∞, *p* = 0.235). The average difference in mean IOP at the individual measurement times between both cohorts was 0.24 mmHg (SD 0.17) on day 1 and 0.44 mmHg (SD 0.6) on day 2. The non-inferiority of PF tafluprost compared to latanoprost regarding fluctuation range could not be demonstrated for both days. **Conclusions:** The data suggests slight inferiority of PF tafluprost to latanoprost eye drops. There were similar mean 24 h IOP values in both cohorts. PF tafluprost eye drops remain a useful treatment option as long as the patient’s individual target pressure is achieved. Furthermore, according to the literature, it is also a treatment option for patients with symptoms of ocular surface disease and other side effects affecting the ocular surface while receiving preservative-containing topical therapy.

## 1. Introduction

Glaucoma is the second most common cause of blindness worldwide, after cataracts. In 2020, 3.6 million people worldwide who were 50 years old or older became blind as a consequence of glaucoma [1].

The main risk factor for glaucoma progression is increased intraocular pressure (IOP). Lowering elevated IOP is still the only possible way to prevent glaucoma progression [2]. To assess IOP, often only one measurement is carried out, usually during normal practice opening hours. Since IOP fluctuates over the course of the day, a diurnal tension profile with several pressure measurements during the day and at night can better reflect the dynamics of IOP compared to individual measurements and thus positively influence the choice of therapy [3,4].

In most cases, first-line therapy is topical IOP-lowering medication as monotherapy [5]. Preparations from different substance classes are available, including prostaglandin analogues [6]. They reduce intraocular pressure by approximately 25% to 35% and have the largest effect on IOP of all classes of substances that can be applied topically [5].

The prostaglandin analogues include latanoprost eye drops (Xalatan^®^) and preservative-free (PF) tafluprost eye drops (Taflotan^®^ sine), among others. The comparison of their effectiveness in reducing IOP is the subject of this trial. Xalatan^®^ contains benzalkonium chloride as a preservative [7]. Benzalkonium chloride is considered the main cause of the development of glaucoma therapy-associated ocular surface disease [8]. Signs and symptoms of ocular surface disease may include ocular discomfort, tear film instability, meibomian gland dysfunction, conjunctival inflammation, subconjunctival fibrosis, epithelial damage, and allergic blepharitis. Konstas et al. report that a large number of clinical and experimental studies have documented a strong correlation of signs and symptoms of ocular surface disease with the number of glaucoma medications used and, in particular, with the cumulative exposure to benzalkonium chloride. The prevalence of ocular surface disease in glaucoma patients is approximately 30–70%, compared to 5–30% in a similar-aged population without glaucoma [9]. The breadth of this spectrum can be explained, for example, by the different number of antiglaucoma medications used and the daily exposure dose of preservatives from local therapy. Furthermore, in ocular surface disease that may already be present before the start of pressure-lowering local therapy, age and hormone status play a role. Additionally, the diagnostic criteria and testing procedures used to determine ocular surface disease are varied. Due to the symptoms caused by benzalkonium chloride, patients’ compliance with their glaucoma therapy may decrease, thereby promoting disease progression. Benzalkonium chloride-free preparations therefore offer an important alternative [8]. Hollo et al. emphasize the importance of preservative-free eye drops in glaucoma therapy [10].

One of the preservative-free substances is PF tafluprost. The effectiveness of latanoprost and PF tafluprost has already been examined and compared in prior studies. Several publications describe a similar effectiveness of both preparations on IOP [11,12,13,14,15,16,17]. Uusitalo et al. detected that when switching from latanoprost to PF tafluprost eye drops, IOP was maintained at the same level, but PF tafluprost was better tolerated in patients who had signs or symptoms of ocular surface disease while on preserved latanoprost [18]. Konstas et al. observed an even greater 24 h efficacy of PF tafluprost compared to preserved latanoprost [19]. To date, studies comparing the effectiveness of PF tafluprost eye drops with latanoprost eye drops in the context of a tension profile over 48 h, including IOP measurements at midnight in a supine position, are missing.

The objective of the present study was to investigate the potential non-inferiority of the efficacy of PF tafluprost eye drops compared to the efficacy of latanoprost eye drops in reducing mean 24 h IOP on the second day of a diurnal IOP profile.

## 2. Materials and Methods

In this retrospective cohort study, patients suffering from primary open-angle glaucoma, ocular hypertension, normal tension glaucoma, pigment dispersion glaucoma, or pseudoexfoliation glaucoma were included and separated into two different cohorts. All patients were required to have had an inpatient stay in the Department of Ophthalmology of the Mainz University Medical Center with diurnal IOP measurement. Measurement times were at 8 a.m./2 p.m./6 p.m./9 p.m. (Goldmann applanation tonometry) and midnight (Perkins tonometer, patient positioned supine). For the midnight measurement, the patients were awakened and the measurement was taken at the patient’s bed in a supine position. At the time of diurnal IOP measurement, patients of cohort 1 had to use latanoprost eye drops as monotherapy for at least 4 weeks in the study eye; patients of cohort 2 had used PF tafluprost eye drops for at least 4 weeks. Patients who had used other IOP-lowering medications within the last 4 weeks were excluded. Further exclusion criteria for the study eye were glaucoma surgery, keratoplasty, laser in situ keratomileusis, angle-closure glaucoma, conditions in the study eye that made applanation tonometry impossible, the use of topical or systemic steroids, and missing midnight measurements during the tension profile.

Neither approval from the ethics committee nor patient informed consent was required for this study, as only routine data from patients who had an inpatient diurnal tension profile in the eye clinic of the Mainz University Medical Center were collected and evaluated retrospectively and the analysis of this study was performed anonymously. This procedure is in accordance with the State Hospital Act (§ 36 and § 37) in Rhineland-Palatinate.

The primary endpoint was to test the potential non-inferiority of the efficacy of PF tafluprost eye drops compared to the efficacy of latanoprost eye drops in reducing mean 24 h IOP on the second day of a diurnal IOP profile.

Secondary endpoints included the investigation of a potential non-inferiority of PF tafluprost eye drops in reducing the mean 24 h IOP on the first day of the diurnal IOP profile, compared to IOP reduction with latanoprost eye drops. Furthermore, the non-inferiority of the efficacy of PF tafluprost eye drops was investigated compared to the efficacy of latanoprost eye drops in reducing the average 24 h fluctuation range on the first and second day of the diurnal IOP profile and in reducing the average 24 h maximum as well as minimum IOP on the first and second day of the diurnal IOP profile.

The following parameters were collected:

- Gender.
- Main diagnosis: primary open-angle glaucoma, ocular hypertension, normal tension glaucoma, pigment dispersion glaucoma, pseudoexfoliation glaucoma.
- Antiglaucomatous topical therapy: PF tafluprost eye drops or latanoprost eye drops.
- Study eye (for statistical analysis, in patients where both eyes were suitable as study eyes, the right eye was selected as the study eye).
- Age at the time of diurnal IOP profile.
- Date of the day the patient was admitted to the diurnal IOP profile.
- Measurement of intraocular pressure (in mmHg) at 8 a.m., 2 p.m., 6 p.m., 9 p.m., and 12:00 a.m. on the day of admission until discharge.
- Lens status of the study eye at the time of the diurnal IOP profile (phakic or pseudophakic).
- Visual acuity at the time of the diurnal IOP profile.
- Central corneal thickness in µm of the study eye.
- Cup-to-disc ratio of the study eye (Heidelberg Retina Tomograph).
- Mean deviation in the visual field examination using the Humphrey Field Analyzer or Octopus perimeter of the study eye.

The data was collected using the SAP operating system (version 7500.1.6.1161) of the Mainz University Medical Center.

The case number for a non-inferiority test was calculated. It was assumed that both therapies were equally effective and that there was a normal distribution of IOP with a standard deviation of 2.4 mmHg in both cohorts. The calculation was carried out for the significance level α = 5% and a power of 80%. A non-inferiority margin of 1.5 mmHg was assumed [20]. This resulted in a case number of 33 patients per cohort, with one eye from each patient being included. The sample size calculation was carried out by the Institute for Medical Biometry, Epidemiology, and Informatics in Mainz (IMBEI). SPSS version 27.0.1.0 was used for statistical data analysis.

For the descriptive statistics of the diurnal IOP profiles, the number of available IOP measurements at each time of day on the first and second day of the diurnal IOP profile was calculated as absolute and percentage frequencies. Next, the IOP values at each measurement time on both days of the IOP profile were described using the mean and standard deviation, as well as the minimum, maximum, and median. The same statistical features were calculated separately for the mean 24 h IOP, the fluctuation range, and the IOP maxima and minima for each of the two days of the diurnal IOP profile. In the case of the mean 24 h IOP, the individual mean 24 h IOP was calculated for each patient based on all five measurement time points. Based on these values, the mean 24 h IOP of the cohorts receiving PF tafluprost and latanoprost was then determined. The individual fluctuation range was defined as the difference between the highest and lowest measured IOP of the five measurements taken per day of the diurnal IOP profile.

SPSS version 27.0.1.0 was also used for inferential statistics. The collected data from the diurnal IOP profiles meet the requirements for performing a one-sided *t*-test or Welch test for independent samples. These tests were used to compare the means. A one-sided 95% confidence interval was determined. The Levene test was used to check the data for homogeneity of variance. If the significance of this test was below 0.2, this indicated unequal variances, and the Welch test was performed. If it was above 0.2, homogeneity of variance could be assumed, and the *t*-test was performed. A non-inferiority threshold of 1.5 mmHg was set to test the data for non-inferiority. Non-inferiority can therefore only be assumed if the *p*-value is less than 0.05 and both the mean difference between latanoprost and PF tafluprost and the one-sided confidence interval do not fall below −1.5 mmHg.

To counteract the accumulation of alpha errors in multiple testing, the Bonferroni–Holm correction was applied to the previously calculated *p*-values. The *p*-values are sorted according to their magnitude, and the smallest *p*-value is multiplied by the total number n of tests performed. The next highest *p*-value is multiplied by n − 1, and the next highest by n − 2. This procedure is repeated with the remaining *p*-values. This yields corrected *p*-values, which can again be assessed using the significance level of 0.05.

## 3. Results

The analyzed diurnal IOP profiles were carried out in the period from 7 January 2005 to 9 July 2019, inclusive.

Ninety-three eyes were included (n = 59 latanoprost group, 24 male, 35 female, average age 63.2 years (SD: 12.2); n = 34 PF tafluprost group, 11 male, 23 female, average age 64.2 years (SD: 9.5)). The mean logMAR visual acuity in the PF tafluprost cohort was 0.1 (SD 0.14); in the latanoprost cohort it was 0.14 (SD 0.26).

In the PF tafluprost cohort, primary open-angle glaucoma was present in 18 cases (53%), normal-tension glaucoma in 8 cases (24%), ocular hypertension in 4 cases (12%), pseudoexfoliation glaucoma in 3 cases (9%), and pigment dispersion glaucoma in 1 case (3%).

In the latanoprost cohort, 28 patients (47%) suffered from primary open-angle glaucoma, 13 patients (22%) from normal-tension glaucoma, 7 patients (12%) from ocular hypertension, 6 patients (10%) from pseudoexfoliation glaucoma, and 5 patients (8%) were affected by pigment dispersion glaucoma.

In the cohort treated with PF tafluprost, the study eye was phakic in 22 (65%) of the total 34 patients and pseudophakic in 12 patients (35%).

Mean central corneal thickness in this cohort was 521.8 μm (SD 30.3), with a maximum of 585 μm and a minimum of 450 μm; the mean cup-to-disc ratio was 0.43 (SD 0.18) with a minimum of 0.04 and a maximum of 0.80.

A total of 14 of the 34 patients in the cohort receiving PF tafluprost underwent a visual field examination using the Humphrey Field Analyzer. The mean of the mean deviation in this subgroup was −2.30 dB (SD 3.77 dB, minimum—11.60 dB, maximum 2.25 dB). In total, 20 of the 34 patients in the cohort receiving PF tafluprost underwent a visual field examination using the Octopus perimeter. The mean of the mean deviation in this subgroup was 3.31 dB (SD 4.62 dB, minimum −1.70 dB, maximum 17.1 dB).

In the cohort treated with latanoprost, there were 53 (90%) patients in whom the study eye was phakic and 6 (10%) in whom it was pseudophakic.

Mean central corneal thickness in this cohort was 523.1 μm (SD 36.0), with a maximum of 617 μm and a minimum of 404 μm; the mean cup-to-disc ratio was 0.45 (SD 0.18, minimum 0.0, maximum 0.8).

A total of 21 of the 59 patients in the cohort receiving latanoprost underwent a visual field examination using the Humphrey Field Analyzer. The mean of the mean deviation in this subgroup was −2.55 dB (SD 6.64 dB). The minimum mean deviation was −21.42 dB, and the maximum was 7.00 dB. Of the 59 patients in the cohort receiving latanoprost, 38 underwent a visual field examination using the Octopus perimeter. The mean of the mean deviation in this subgroup was 4.67 dB (SD 4.92 dB). The minimum mean deviation was −1.30 dB, and the maximum was 18.30 dB.

Table 1 shows the distribution of IOP values in the two cohorts on day 1 of the diurnal IOP profile.

**Figure 1:**
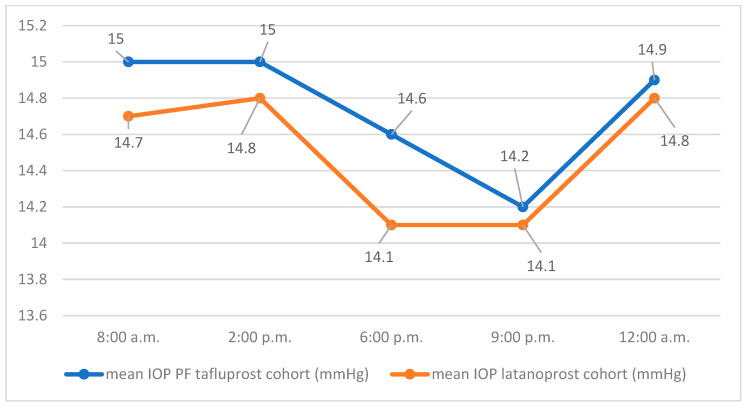
Mean IOP values for the respective measurement times on day 1.

The course of the mean IOP values of both groups at the respective measurement times on day 1 is shown in Figure 1.

The mean 24 h IOP of the latanoprost cohort on day 1 of the IOP profile was 14.5 mmHg (SD 2.6) with a minimum of 9.7 mmHg, a maximum of 23 mmHg, and a median of 14.4 mmHg. The mean 24 h IOP of the cohort using PF tafluprost was 14.7 mmHg (SD 3.5) with a minimum of 9.3 mmHg, a maximum of 25 mmHg, and a median of 13.8 mmHg. The average difference in mean values of 24 h IOP (latanoprost, PF tafluprost) was −0.24 mmHg (SD 0.17) on day 1. Using a one-sided Welch test, the hypothesis of non-inferiority of PF tafluprost eye drops to latanoprost eye drops in efficacy on mean 24 h IOP could not be confirmed for day 1 (95% CI −1.3, ∞, *p* = 0.165).

Table 2 shows the distribution of IOP values in the two cohorts on day 2 of the diurnal IOP profile.

**Figure 2:**
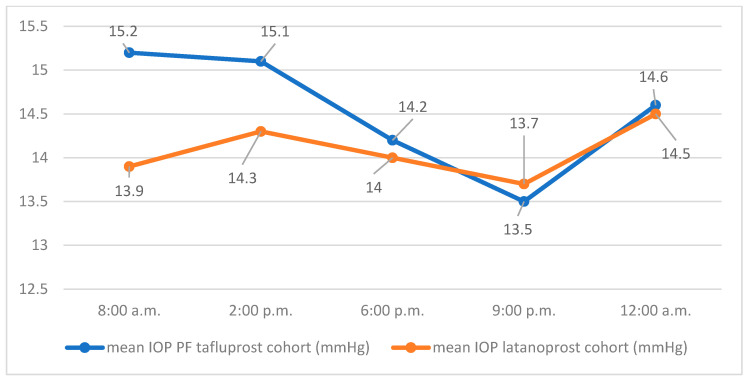
Mean IOP values at the respective measurement times on day 2.

The course of the mean IOP values of both groups at the respective measurement times on day 2 is shown in Figure 2.

On day 2, the mean 24 h IOP was 14.1 mmHg (SD 2.3) for the latanoprost group with a minimum of 10 mmHg, a maximum of 22.4 mmHg, and a median of 14.4 mmHg. The mean 24 h IOP of the PF tafluprost group on day 2 was 14.5 mmHg (SD 3.4) with a minimum of 9.4 mmHg, a maximum of 23.4 mmHg, and a median of 13.8 mmHg. The average difference in mean values of 24 h IOP (latanoprost, PF tafluprost) was −0.44 mmHg (SD 0.6) on day 2. Using a one-sided Welch test, the hypothesis of non-inferiority of PF tafluprost eye drops to latanoprost eye drops in efficacy on mean 24 h IOP could not be confirmed for day 2 (95% CI −1.5, ∞, *p* = 0.235).

On day 1, the fluctuation range of the IOP in the PF tafluprost group had a minimum of 1 mmHg, a maximum of 11 mmHg, a mean of 5.1 mmHg (SD 2.9), and a median of 4.5 mmHg. In the latanoprost group, the fluctuation range had a minimum of 1 mmHg, a maximum of 10 mmHg, a mean of 4.3 mmHg (SD 2.2), and a median of 4.0 mmHg. The average difference in the mean values of fluctuation range (latanoprost-PF tafluprost) was −0.8 mmHg. The Welch test showed no statistical significance for the mean of the IOP fluctuation range of both groups on day 1 (95% CI −1.7, ∞, *p* = 0.314).

On day 2, the fluctuation range of the IOP in the PF tafluprost group had a minimum of 2 mmHg, a maximum of 18 mmHg, a mean of 5.7 mmHg (SD 3.1), and a median of 5.0 mmHg. In the latanoprost group, the fluctuation range had a minimum of 2 mmHg, a maximum of 15 mmHg, a mean of 4.9 mmHg (SD 2.2), and a median of 5.0 mmHg. The average difference in the mean values of fluctuation range (latanoprost-PF tafluprost) was −0.8 mmHg. The Welch test showed no statistical significance for the mean of the IOP fluctuation range of both groups on day 2 (95% CI −1.8, ∞, *p* = 0.236).

On day 1, the mean of the IOP maxima in the PF tafluprost group was 17.2 mmHg (SD 4.3), with a minimum of 11 mmHg, a maximum of 27 mmHg, and a median of 16 mmHg. In the latanoprost group, the mean of the IOP maxima was 16.8 mmHg (SD 3.1), with a minimum of 10 mmHg, a maximum of 26 mmHg, and a median of 17 mmHg. The average difference in the mean values of the IOP maxima on day 1 (latanoprost-PF tafluprost) was −0.4 mmHg. The Welch test shows no statistical significance for the mean of the IOP maxima of both groups on day 1 (95% CI −1.7, ∞, *p* = 0.344).

On day 2, the mean of the IOP maxima in the PF tafluprost group was 17.7 mmHg (SD 4.7), with a minimum of 10 mmH, a maximum of 32 mmHg, and a median of 17.5 mmHg. In the latanoprost group, the mean of the IOP maxima was 16.7 mmHg (SD 3.2) with a minimum of 11 mmHg, a maximum of 27 mmHg, and a median of 16 mmHg. The average difference in the mean values of the IOP maxima (latanoprost—PF tafluprost) on day 2 was 1.0 mmHg. The Welch test shows no statistical significance for the mean of the IOP maxima of both groups on day 2 (95% CI −2.5, ∞, *p* = 0.284).

On the first day of the diurnal IOP profile, the mean of the IOP minima in the PF tafluprost group was 12.1 mmHg (SD 3.2), with a minimum of 6 mmHg, a maximum of 24 mmHg, and a median of 12 mmHg. In the latanoprost group, the mean of the IOP minima was 12.5 mmHg (SD 2.5) with a minimum of 8 mmHg, a maximum of 19 mmHg, and a median of 12 mmHg. The average difference in the mean values of the IOP minima on day 1 (latanoprost-PF tafluprost) was 0.4 mmHg. The one-tailed *t*-test showed statistical significance for the mean of the IOP minima of both groups on day 1 (95% CI −0.6, ∞, *p* = 0.008). With regard to the mean of the IOP minima on day 1, there is therefore non-inferiority of PF tafluprost compared to latanoprost.

On day 2, the mean of the IOP minima in the PF tafluprost group was 12.0 mmHg (SD 2.8), with a minimum of 8 mmHg, a maximum of 22 mmHg, and a median of 11.5 mmHg. In the latanoprost group, the mean of the IOP minima was 11.8 mmHg (SD 2.3) with a minimum of 8 mmHg, a maximum of 18 mmHg, and a median of 12 mmHg. The average difference in the mean values of the IOP minima on day 2 (latanoprost-PF tafluprost) was −0.2 mmHg. The Welch test showed no statistical significance for the mean of the IOP minima of both groups on day 2 (95% CI −1.1, ∞, *p* = 0.06). Table 3 summarizes the results of the analytical statistics of the diurnal IOP profiles.

## 4. Discussion

To date, there are no studies in the current literature in which a comparison of the effectiveness of PF tafluprost eye drops and latanoprost eye drops was carried out based on inpatient diurnal IOP profiles over more than 24 h including IOP measurements at midnight in a supine position. The aim of this study was to investigate this.

To achieve a power of 80% and a significance level of 5%, the sample size calculation was based on the assumption that there was a normal distribution of IOP with a standard deviation of 2.4 mmHg in both cohorts, as well as both prostaglandin analogues being equally effective. On day two of the tension profile, the standard deviation of IOP in the group receiving PF tafluprost eye drops was 3.4 mmHg. The standard deviation assumed for the sample size calculation is therefore significantly lower than the standard deviation determined. In the latanoprost cohort, there was a standard deviation of IOP of 2.3 mmHg, a slightly lower value than the standard deviation assumed at the start. The higher standard deviation in the cohort receiving PF tafluprost eye drops reduces the real power of this study, although the slightly lower standard deviation in the group receiving latanoprost eye drops slightly increases the power.

A non-inferiority study of PF tafluprost eye drops versus latanoprost eye drops by Konstas et al. used a standard deviation of 2.8 mmHg for their case number calculation [13]. A standard deviation of 2.4 mmHg assumed in this study appears to have been an overly optimistic estimate.

A sample size of 33 patients was calculated for both cohorts. A total of 34 patients were included in the PF tafluprost group and 59 patients in the latanoprost group. The power can also be improved due to the higher number of patients. With a larger number of subjects, outliers in IOP (both high and low) in the diurnal measurements have less of an impact on the mean value than with a smaller number of subjects. The points mentioned should be taken into account critically when interpreting the results.

With regard to the patient population, the following consideration should also be factored in: diurnal IOP profiles are only carried out on patients who have had abnormalities during outpatient ophthalmological examination. Patients in whom the IOP is well controlled under monotherapy and who show no evidence of progression are not subjected to a diurnal IOP profile. In this respect, the patients in this study do not represent a ubiquitously representative proportion of the total number of glaucoma patients treated with PF tafluprost eye drops or latanoprost eye drops. It is conceivable that the intraocular pressure values recorded may be higher for this reason than in the overall population not included in this study. Had the measurement parameters been collected within the framework of a prospective study, there would have been better representativeness compared to reality. Since, retrospectively, it was no longer apparent in many cases what the exact indication for the inpatient diurnal IOP profile was, this information could not be obtained. With mean maximum IOP values on the first day of the diurnal IOP profile of 17.2 mmHg (SD 4.3 mmHg) and a maximum of 27 mmHg in the cohort under PF tafluprost, and mean maximum IOP values of 16.8 mmHg (SD 3.1 mmHg) and a maximum of 26 mmHg in the cohort under latanoprost, it can be assumed that in some patients, the indication for the diurnal IOP profile was insufficiently controlled intraocular pressure.

The information as to whether a change in therapy took place after carrying out the IOP profile was not collected in this study, but it would be informative, as a change would indicate an individually inadequate effectiveness of PF tafluprost eye drops or latanoprost eye drops.

Another difficulty is that we do not know the baseline IOP without local pressure-lowering medication, and, therefore, no statement can be made about the extent of the pressure reduction caused by the therapy used. Patients exhibiting a higher baseline IOP tend to also exhibit higher IOP levels while on medication compared to patients with a lower baseline IOP [17]. Future analyses should also include a separate evaluation of the subgroups, such as NTG and OHT.

When interpreting the data, it should be noted that some of the measurements were performed by different examiners: from 6 p.m. onward, the measurements were performed not by the ward physician but by the on-duty ophthalmologist. Furthermore, the previous measurements were visible to each examiner. Consequently, inter- and intra-observer variability also may have influenced the recorded values; however, in this highly specialized glaucoma clinic, all physicians have great expertise in IOP measurement. It should be noted that IOP measurements over 48 h are available only at very few clinics.

During the inpatient stay for the IOP profile, some patients may have experienced temporary medication-induced mydriasis, including cycloplegic effects. This was insufficiently documented in some cases and therefore could not be meaningfully assessed. The medication-induced mydriasis may have also influenced the measured IOP values, but this factor applied to both groups.

Only patients without any glaucoma surgery in the past were included in this study, so that the effectiveness of PF tafluprost eye drops and latanoprost eye drops on intraocular pressure could be assessed without the influence of surgical IOP-lowering measures to reduce pressure, as Lim et al. showed that even 20 years after a trabeculectomy, the IOP was below 21 mmHg in 57% of patients without further medication and the operation was still affecting the IOP [21].

A major strength and peculiarity of this study is that IOP values collected during an inpatient stay of over 48 h were analyzed. The IOP values of the first day of the diurnal pressure profile likely reflect the patients’ actual values in their everyday lives outside the hospital more accurately, as the patients had to self-administer the eye drops the evening before their hospital admission. In the hospital setting, correct application is ensured by nursing staff. However, since the focus of this study is on comparing the efficacy of PF tafluprost and latanoprost, the primary endpoint requires that the eye drops be administered correctly at the prescribed time. For this reason, the second day of the diurnal IOP profile is evaluated for this purpose. Nevertheless, an inpatient diurnal IOP profile, during which five measurements are taken per day, cannot fully capture the actual circadian dynamics of the patient’s IOP in their normal out-of-hospital environment. Patients cannot, for example, engage in sporting activities they would normally pursue while in the hospital. Consequently, a possibly hypotensive effect on IOP is absent.

The primary endpoint of this study was to assess whether PF tafluprost eye drops are non-inferior in efficacy compared to latanoprost eye drops in terms of the 24 h mean IOP on the second day of the diurnal IOP profile. This hypothesis could not be confirmed using a one-sided Welch test (latanoprost 14.1 mmHg SD 2.3 versus PF tafluprost 14.5 mmHg SD 3.4; 95% CI −1.5, ∞, *p* = 0.235). The result did not show statistical significance, and, therefore, the primary endpoint of this study could not be achieved. Nevertheless, based on the descriptive statistics, it can be stated that a similar mean 24 h IOP was found in the two cohorts on the second day of the diurnal IOP profile. The difference (−0.4 mmHg) between the two cohorts is of questionable clinical relevance.

One of the secondary endpoints of this study was the assessment of the non-inferiority of PF tafluprost eye drops compared to latanoprost eye drops on the 24 h mean IOP of the first day of the diurnal IOP profile. Here, similarly, the hypothesis could not be confirmed. There was no statistically significant result (latanoprost 14.5 mmHg SD 2.6 versus PF tafluprost 14.7 mmHg SD 3.5; 95% CI −1.3, ∞, *p* = 0.165), although the difference between the mean 24 h IOP on the first day between latanoprost and PF tafluprost was even lower compared to the second day (−0.2 mmHg versus −0.4 mmHg). However, on both days of the IOP profile, the standard deviation of the cohort receiving PF tafluprost eye drops was significantly higher than that of the cohort receiving latanoprost eye drops. Since the standard deviation depends, among other things, on the number of cases, it would be possible that if the number of cases in the cohort receiving PF tafluprost eye drops was the same as in the cohort receiving latanoprost eye drops, there would be a comparable standard deviation in both cohorts.

Konstas et al. conducted a study in which patients affected by primary open-angle glaucoma exhibiting a mean 24 h IOP of 16–17 mmHg or less showed no evidence of glaucoma progression within five years in 75% of cases. Since the mean 24 h IOP in our study was significantly below this range on both days of the diurnal IOP profile in both cohorts, it can be assumed that a high percentage of the patients in our cohorts will also not experience any progression within five years. There were also patients in this study whose mean 24 h IOP was above 16–17 mmHg. For them, this statement cannot be generalized and the progression rate is probably higher in this case. However, these assumptions remain speculative for the patients in this trial due to the retrospective study design. It should also be noted that, in contrast to the study by Konstas et al., this study also includes other types of glaucoma in addition to primary open-angle glaucoma [22]. In their study, Konstas et al. further reported a similar efficacy of PF tafluprost eye drops and latanoprost eye drops over 24 h, without any statistically significant differences between the two prostaglandin analogues. The mean 24 h IOP was thereby 17.8 mmHg (SD 2.2 mmHg) under therapy with PF tafluprost and 17.7 mmHg (SD 2.1) under latanoprost, which, compared to our results, was notably higher in both cohorts. In contrast to our study, additional nighttime pressure measurement using a Perkins tonometer slightly increases the significance of the mean 24 h intraocular pressure in the study by Konstas et al. compared to this study.

In another study by Konstas et al. with insufficiently controlled IOP under therapy with latanoprost eye drops (22.2 mmHg, SD 2.9), a statistically significant reduction in mean 24 h IOP was observed after switching to therapy with PF tafluprost eye drops (21.9 mmHg, SD 3.2) [19]. The difference between the mean 24 h IOP in the PF tafluprost cohort and the latanoprost cohort is in a similar range on both the first and second day of the diurnal tension profile in this study.

Ranno et al. further reported no statistically significant differences in the mean IOP when comparing patients treated with PF tafluprost (16.6 mmHg, SD 2) and latanoprost (16.5 mmHg, SD 2.3). In contrast to our study, there was no nighttime pressure measurement. Only measurements between 8:00 a.m. and 8:00 p.m. were performed, which reduces the significance of the IOP profile [12]. Significant fluctuations could therefore have been overlooked during this period [23].

The individual circadian fluctuation range of the IOP in healthy eyes has a maximum of 6 mmHg; in glaucoma patients, it often exceeds 10 mmHg [24]. Since the fluctuation range represents a potential risk factor for glaucoma progression, knowledge of this parameter is of great importance. Prostaglandin analogues reduce the fluctuation range more than other antiglaucoma drugs [25]. In our study, there was a difference in the mean fluctuation range of −0.8 mmHg on the first and second day of the diurnal IOP profile between the cohort receiving latanoprost and the cohort receiving PF tafluprost. When testing for non-inferiority of PF tafluprost versus latanoprost in terms of mean fluctuation range using a one-sided Welch test, no statistically significant results were observed for both days. Therefore, it cannot be assumed that PF tafluprost eye drops are non-inferior in comparison to latanoprost eye drops in reducing the mean fluctuation range of the IOP. This secondary endpoint was not met. Nevertheless, the data shows that in this study, the mean fluctuation range on the first and second days of the tension profile for patients in both cohorts is within a range that would also be similar for people not affected by glaucoma [24].

The non-inferiority of PF tafluprost eye drops to latanoprost eye drops in terms of mean maximum pressure could not be demonstrated for either the first or the second day. This secondary endpoint is therefore not reached.

For the first day of the IOP profile, the non-inferiority of PF tafluprost eye drops compared to latanoprost eye drops for the mean minimum pressure was demonstrated using a one-sided *t*-test. This finding was not confirmed for the second day; the result only just did not reach statistical significance, but the values in both cohorts were very close to one another, with a difference of −0.2 mmHg for latanoprost versus PF tafluprost. Of all the pre-formulated endpoints, only the secondary endpoint related to the pressure minimum on the first day of the IOP profile could be achieved. There is no reproducibility of this result for the second day of the IOP profile, which underlines the relevance of an IOP profile beyond a period of 24 h.

In the literature, varying outcomes are reported when comparing the effectiveness of the two prostaglandin analogues on the IOP by the assessment of different parameters. The spectrum ranges from a comparable effectiveness of the two preparations to a statistically significant superior effectiveness of PF tafluprost eye drops on the IOP compared to latanoprost eye drops. However, most studies show similar effectiveness of PF tafluprost eye drops and latanoprost eye drops [11,12,13,14,15,16,17].

## 5. Conclusions

Based on the data from our study, for the most part, it cannot be assumed that PF tafluprost eye drops are non-inferior to latanoprost eye drops. In particular, the mean 24 h IOP on the first and second day of the IOP profile, the mean IOP maxima on the first day, the mean IOP minima on the first and second day, as well as the mean IOP difference between the two cohorts are of questionable clinical relevance. Nevertheless, PF tafluprost eye drops are an effective therapy option as long as the IOP of the treated glaucoma patients is within the individual target range during therapy.

The research in the literature shows that PF tafluprost eye drops can also represent a treatment option for patients with symptoms of ocular surface disease and other side effects affecting the ocular surface following local therapy containing preservatives [11,14,19,26].

Future prospective studies with a crossover design could provide further important information on the comparability of the two preparations.

## Figures and Tables

**Table 1:** IOP day 1 of the diurnal IOP profile per cohort.

Timepoint of IOP Measurement Day 1	Cohort	Mean IOP (SD) (in mmHg)	Minimum IOP (in mmHg)	Maximum IOP (in mmHg)	Median IOP (in mmHg)
8:00 a.m.	PF tafluprost	15.0 (3.3)	12.0	24.0	14.0
8:00 a.m.	latanoprost	14.7 (3.5)	10.0	20.0	16.0
2:00 p.m.	PF tafluprost	15.0 (4.0)	9.0	24.0	14.0
2:00 p.m.	latanoprost	14.8 (3.0)	8.0	26.0	14.0
6:00 p.m.	PF tafluprost	14.6 (4.1)	7.0	25.0	14.0
6:00 p.m.	latanoprost	14.1 (3.1)	9.0	23.0	14.0
9:00 p.m.	PF tafluprost	14.2 (3.8)	10.0	27.0	13.3
9:00 p.m.	latanoprost	14.1 (2.9)	8.0	20.0	14.0
12:00 a.m.	PF tafluprost	14.9 (4.7)	6.0	26.0	14.0
12:00 a.m.	latanoprost	14.8 (3.4)	8.0	24.0	14.0

**Table 2:** IOP day 2 of the diurnal IOP profile per cohort.

Timepoint of IOP Measurement Day 2	Cohort	Mean IOP (SD) (in mmHg)	Minimum IOP (in mmHg)	Maximum IOP (in mmHg)	Median IOP (in mmHg)
8:00 a.m.	PF tafluprost	15.2 (4.9)	10.0	32.0	14.0
8:00 a.m.	latanoprost	13.9 (2.5)	8.0	22.0	14.0
2:00 p.m.	PF tafluprost	15.1 (4.3)	10.0	27.0	14.0
2:00 p.m.	latanoprost	14.3 (3.4)	8.0	27.0	14.0
6:00 p.m.	PF tafluprost	14.2 (4.0)	8.0	26.0	14.0
6:00 p.m.	latanoprost	14.0 (3.4)	8.0	26.0	14.0
9:00 p.m.	PF tafluprost	13.5 (3.4)	8.0	23.0	13.0
9:00 p.m.	latanoprost	13.7 (2.9)	9.0	23.0	14.0
12:00 p.m.	PF tafluprost	14.6 (3.7)	8.0	24.0	14.0
12:00 p.m.	latanoprost	14.5 (3.0)	8.0	26.0	15.0

**Table 3:** Results of the analytical statistics of the diurnal IOP profiles.

Parameter	Latanoprost (SD) (in mmHg)	PF Tafluprost (SD) (in mmHg)	Mean Difference (Latanoprost—PF Tafluprost) (in mmHg)	One-Sided 95%- Confidence- Interval	*p*-Value *
Mean average 24 h IOP Day 1 ^✕^	14.5 (2.6)	14.7 (3.5)	−0.2	−1.3, ∞	0.165
Mean fluctuation range day 1 ^✕^	4.3 (2.2)	5.1 (2.9)	−0.8	−1.7, ∞	0.314
Mean IOP maxima day 1 ^✕^	16.8 (3.1)	17.2 (4.3)	−0.4	−1.7, ∞	0.344
Mean IOP minima day 1 ^△^	12.5 (2.5)	12.1 (3.2)	0.4	−0.6, ∞	0.008
Mean average 24 h IOP Day 2 ^✕^	14.1 (2.3)	14.5 (3.4)	−0.4	−1.5, ∞	0.235
Mean fluctuation range day 2 ^✕^	4.9 (2.2)	5.7 (3.1)	−0.8	−1.8, ∞	0.236
Mean IOP maxima day 2 ^✕^	16.7 (3.2)	17.7 (4.7)	−1.0	−2.5, ∞	0.284
Mean IOP minima day 2 ^✕^	11.8 (2.3)	12.0 (2.8)	−0.2	−1.1, ∞	0.060

* Bonferroni–Holm corrected *p*-values; *p* < 0.05: non-inferiority of PF tafluprost compared to latanoprost; ✕: Welch test; △: *t*-test.

## Data Availability

The raw data supporting the conclusions of this article will be made available by the authors on request.
